# Feminizing Adrenocortical Carcinoma in Men: A Rare Cause of Persistent Gynecomastia and a Contemporary Literature Review

**DOI:** 10.3390/epidemiologia6040064

**Published:** 2025-10-15

**Authors:** Ana Maria Arnautu, Diana Loreta Paun, Corina Neamtu, Costin Gingu, Victor Nimigean, Dana-Mihaela Tilici, Ruxandra Costinescu, Mirona Costea, Adina Onofrei, Beatrice Grecu, Claudia Nacea-Radu, Sorin Paun

**Affiliations:** 1 Endocrinology Department, Bucharest University Emergency Hospital, 050098 Bucharest, Romania; ana-maria.arnautu@rez.umfcd.ro (A.M.A.); ruxandracostinescu@gmail.com (R.C.); beatrice-mihaela.grecu@rez.umfcd.ro (B.G.);; 2 Faculty of Medicine, University of Medicine and Pharmacy “Carol Davila”, 020021 Bucharest, Romania; victor.nimigean@umfcd.ro (V.N.); sorin.paun@umfcd.ro (S.P.); 3 Clinic Hospital SANADOR, 010997 Bucharest, Romania; 4 “Fundeni” Clinical Institute, 022328 Bucharest, Romania; 5 Doctoral School, University of Medicine and Pharmacy “Carol Davila”, 020021 Bucharest, Romania; 6 Bucharest Clinical Emergency Hospital, 014461 Bucharest, Romania

**Keywords:** feminizing adrenocortical carcinoma, bilateral gynecomastia, estrogen-secreting tumor, fertility, low-risk ACC

## Abstract

Background: Feminizing adrenocortical tumors (FATs) are an exceedingly rare subset of adrenal neoplasms, typically affecting adult men and characterized by an excess of estrogen, suppressed gonadotropins, and gynecomastia. Most FATs are malignant, with a poor prognosis and a high risk of recurrence. Case Presentation: We report the case of a 24-year-old male with bilateral gynecomastia, abdominal mass symptoms, and one year of unexplained infertility. A hormonal evaluation revealed elevated estradiol (90.1 pg/mL) and suppressed ACTH (2.6 pg/mL), with inappropriately normal cortisol levels (12.1 µg/dL). Imaging identified a right adrenal mass. The patient underwent open adrenalectomy, and histopathology confirmed stage II adrenocortical carcinoma (T2NxM0) with autonomous estradiol secretion, negative margins, and a Ki-67 index of 10%. Postoperatively, gonadal function normalized, and infertility resolved at two months. The multidisciplinary tumor board considered but did not initiate adjuvant mitotane, given the completely resected low-stage disease. Conclusions: This case illustrates the rare presentation of feminizing adrenocortical carcinoma with reversible infertility and highlights the importance of early recognition and close surveillance. In addition, our literature review of 12 male cases reported between 2015 and 2025 emphasizes gynecomastia as the hallmark presentation and discusses emerging evidence supporting active surveillance as a potential alternative to adjuvant mitotane in selected low-risk patients.

## 1. Introduction

Primary malignancies of the adrenal gland are exceedingly uncommon. Adrenocortical carcinomas (ACCs) are uncommon malignancies, with a global incidence of approximately 2 cases per million individuals annually, while pheochromocytomas and paragangliomas have an incidence of 5–8 cases per million annually [1,2]. These tumors display a bimodal age distribution, with the first incidence peak occurring in early childhood (under the age of 5) and a second peak typically observed during the fourth to fifth decades of life [3]. Adrenocortical tumors may be either functional or non-functional. Functional tumors are classified according to their predominant hormonal secretion. Cortisol-secreting tumors cause Cushing’s syndrome, whereas aldosterone-secreting tumors lead to primary hyperaldosteronism (Conn’s syndrome) [4]. Both adrenal adenomas and adrenocortical carcinomas may produce mineralocorticoids. Less commonly, tumors may secrete androgens, estrogen, or mixed hormonal profiles. Among these, feminizing adrenocortical tumors (FATs), characterized by estrogen overproduction and suppressed gonadotropins, represent an exceptionally rare entity [5].

Feminizing adrenocortical tumors are a rare subset of functional adrenal cortical neoplasms that produce only estrogen, specifically estradiol and estrone. These tumors can present as either benign estrogen-producing adrenal adenomas or, more frequently, as malignant estrogen-secreting adrenocortical carcinomas. Excess estrogen typically leads to the suppression of testosterone production. FATs are extremely uncommon, comprising under 2% of all adrenal tumors [5,6]. Between 1970 and 2015, only 50 cases were reported in the literature [7], with only a few additional cases documented since then.

Presenting signs/symptoms in adult males commonly include gynecomastia, hypogonadism, and weight fluctuation (either loss or gain, with loss being more common); in adult females, there is irregular or postmenopausal bleeding; in pediatric males, there is contrasexual precocious pseudopuberty; and in pediatric females, there is isosexual precocious puberty [8]. In pure FATs, there is usually a lack of signs or symptoms of Cushing syndrome, hypertension, and virilization/hirsutism, differentiating FATs from mixed functional adrenocortical tumors. Diagnosis is made by a combination of clinical factors (including gynecomastia, change in libido, and erectile dysfunction in adult males), serum laboratory values (including elevated estrogen), radiologic imaging findings (i.e., a visualized adrenal mass), and immunohistochemistry (IHC) imaging findings (i.e., α-aromatase positivity). Radiologically and histologically, estrogen-secreting adrenocortical carcinomas (ES-ACCs) appear broadly similar to other primary adrenal masses with the exception of α-aromatase positivity for FATs [5]. Distinguishing malignant from benign FATs is challenging, but can be assisted with radiographic findings (i.e., irregular borders, heterogeneity, invasion, etc.), histology (i.e., a high Weiss score), or recurrence/metastases.

Adrenal adenomas are known to have an excellent prognosis. In contrast, adrenocortical carcinomas are associated with reduced survival rates, and among them, feminizing adrenocortical tumors carry the poorest prognosis. Additionally, advanced disease stage at the time of diagnosis is a strong predictor of unfavorable clinical outcomes. The overall 5-year survival rate for ACCs is approximately 30–40%, but prognosis is highly stage-dependent, ranging from 60 to 80% for localized disease (stage I–II) to less than 20% for advanced disease (stage IV) [9,10].

Although no definitive causal mutations have been identified in feminizing adrenocortical tumors, several genetic alterations have been implicated, including dysregulation of IGF2 expression, aberrations in the Wnt/β-catenin signaling pathway, and mutations in the *TP53.1* gene [11]. Some authors have discussed mutations in tumor suppressor genes [7,10].

While some adrenocortical carcinomas have been associated with hereditary syndromes such as multiple endocrine neoplasia type 1 (MEN1), Carney complex, Beckwith–Wiedemann syndrome, and Li-Fraumeni syndrome, many cases appear to occur sporadically [1]. In the context of feminizing adrenocortical tumors, aside from a single reported case linked to MEN1, genetic investigations have not yet clarified a definitive etiology [12]. These tumors are typically marked by elevated aromatase activity, although in some cases, the increase is only moderate, despite the presence of significant hyperestrogenism.

## 2. Materials and Methods

For the case presentation, written informed consent has been obtained from the patient to publish this paper.

A comprehensive literature search was conducted to identify relevant studies on FATs, focusing on symptoms, treatment strategies, and outcomes. The search strategy encompassed electronic databases and academic repositories from June 1993 to May 2025. The following databases were utilized: PubMed/MEDLINE, Scopus, Web of Science, and Embase.

The search strategy combined Medical Subject Headings (MeSH) terms and free-text keywords, including but not limited to: “Feminizing adrenal carcinoma”, “Infertility due to adrenocortical carcinoma with autonomous estradiol secretion”, “Gynecomastia due to feminizing adrenal tumor”, “Hyperestrogenism”, “Estrogen secreting tumors”, “Low-risk ACC”, “Treatment outcomes”, “Mitotane”, “Patient outcomes”.

The search paradigm included combinations of Boolean operators (AND, OR) to refine the search results. Example search strings used were: “Feminizing adrenal carcinoma” OR “Feminizing adrenal tumor “AND “treatment” OR “mitotane”, “low-risk adrenocortical carcinoma” OR “low-risk ACC”.

To ensure relevance and specificity, the PICO (Population, Intervention, Comparison, Outcome) framework was applied. Inclusion criteria encompassed peer-reviewed articles, reviews, and observational studies published in English. Exclusion criteria included non-English articles, letters to the editor, and studies not directly related to feminizing adrenal carcinoma treatment or outcomes. Case reports were included due to the rarity of the condition and their relevance to clinical presentation and outcomes.

Two independent reviewers screened titles and abstracts for relevance. Full-text articles meeting the inclusion criteria underwent a detailed review. Data extraction included study design, participant characteristics, interventions, outcomes, and key findings.

## 3. Results

### Case Presentation

We present the case of a 24-year-old male diagnosed with adrenocortical carcinoma associated with bilateral gynecomastia due to tumor-derived estrogen secretion.

The disease onset was marked by right upper quadrant abdominal discomfort, reported approximately one month before surgical intervention. The patient also reported progressive bilateral breast enlargement noticed about five months earlier and a history of unexplained infertility over the previous year.

On physical examination, the patient had a normal body mass index and was otherwise unremarkable, except for bilateral symmetric gynecomastia. The testes were of normal size and volume, without palpable nodules or atrophy. No abdominal masses were palpable on clinical examination.

An extensive endocrine evaluation was conducted. The results revealed inappropriately normal cortisol levels (12.1 µg/dL) in the presence of suppressed ACTH (2.6 pg/mL) and significantly elevated estradiol (90.1 pg/mL). Aldosterone, renin, metanephrines, and normetanephrines were within normal limits.

Abdominal ultrasound revealed a nodular mass superior to the right kidney. Further imaging with contrast-enhanced abdominal MRI revealed a well-defined, macronodular right adrenal mass measuring 8.2 × 6 × 5.8 cm, exhibiting internal areas of necrosis and progressive gadolinium enhancement. The lesion was in contact with the superior pole of the right kidney (without evidence of invasion), the inferior vena cava over a 3.4 cm segment, hepatic segments VI and VII, the right diaphragmatic crus, and the duodenum, without a clearly defined plane of separation.

Bilateral breast ultrasound showed retroareolar fibroglandular tissue in a florid phase: right breast with a maximal thickness of 1 cm and length of approximately 2 cm; left breast with a thickness of 1 cm and length of approximately 1.6 cm.

Based on the patient’s clinical presentation, hormonal profile, and imaging findings, a diagnosis of right-sided adrenocortical carcinoma was established.

**Figure 1:**
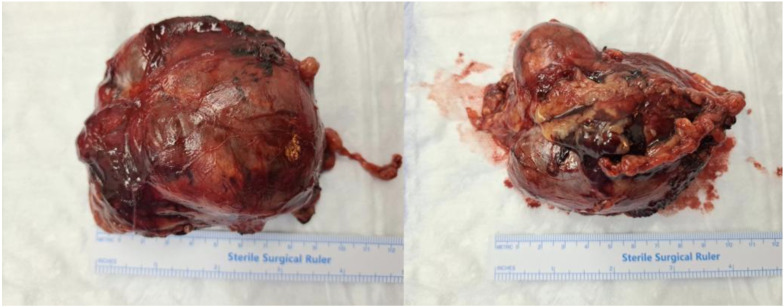
Gross appearance of the resected adrenal tumor. The mass was a large, solitary, and circumscribed tumor (9.5 × 7 × 3.5 cm).

Surgical management involved a right adrenalectomy (Figure 1).

Histopathological evaluation confirmed conventional adrenocortical carcinoma, weighing 162 g and measuring 9.5 × 7 × 3.5 cm. Weiss criteria assessment revealed five of nine criteria, and the patient was diagnosed with a malignant tumor: a Fuhrman nuclear grade of 4, mitotic index of 8 mitoses/50 HPF, presence of atypical mitoses, tumor necrosis, and diffuse architecture with loss of the reticulin framework.

Immunohistochemistry showed a Ki-67 index of 10%, weak cytoplasmic synaptophysin staining in rare tumor cells, Melan-A positivity, granular cytoplasmic inhibin positivity in rare cells, cytoplasmic and focal nuclear calretinin positivity, and p53 positivity in <1% of tumor cells.

Based on histopathological and immunohistochemical findings, the diagnosis was confirmed as stage II adrenocortical carcinoma (T2, Nx, M0) with autonomous estradiol secretion.

Postoperative evaluation at one month revealed no significant clinical changes except for persistent bilateral gynecomastia. Hormonal reassessment demonstrated normalization of the hypothalamic–pituitary–adrenal axis: ACTH 61.8 pg/mL, serum cortisol 10.1 µg/dL, and estradiol decreased to 33.7 pg/mL (over 50% reduction from baseline). DHEA-S, androstenedione, aldosterone, and renin levels were all within normal ranges.

Therapeutic options, including the initiation of adjuvant mitotane therapy, were discussed in detail. Postoperatively, a multidisciplinary team was assembled to evaluate the initiation of mitotane therapy. However, based on the most recent clinical data, the Ki-67 index of 10%, and the patient’s preferences, the patient expressed a preference to defer systemic treatment temporarily in consideration of family planning; it was decided to defer adjuvant treatment for the time being. Follow-up imaging with abdominal–pelvic MRI and thoracic CT was scheduled every 3 months during the first two years, then every 6 months over the next three years in the absence of recurrence.

At the three-month postoperative follow-up, gynecomastia persisted. Breast ultrasound indicated a stable glandular appearance: right breast retroareolar gland measuring 15 × 7 mm and left breast 5 × 10 mm, both in a proliferative phase. Follow-up abdominal–pelvic MRI did not reveal any suspicious tumor recurrence.

Hormonal profile showed elevated ACTH 114 pg/mL, cortisol within the normal range 19.9 µg/dL, normal DHEA-S 128 µIU/mL, and a normalized gonadal axis: LH 1.2 µIU/mL, FSH 2.7 µIU/mL, with testosterone at the lower limit of normal 3.1 ng/mL and estradiol 24.2 pg/mL (Table 1).

## 4. Discussion

This case exhibits several atypical features, including right upper-quadrant abdominal pain, most likely related to the large size of the adrenal mass, although the patient was of normal weight. On clinical examination, bilateral gynecomastia was also identified, which the patient reported noticing approximately five months earlier. Regarding paraclinical evaluations, estradiol levels had normalized by three months postoperatively. Although preoperative gonadotropin levels are not available, the most recent assessment showed low-normal FSH and LH levels, suggesting that these were likely suppressed preoperatively due to estrogen hypersecretion by the tumor.

Feminizing adrenocortical carcinomas are typically sizable masses and become palpable in approximately 62% of cases at advanced stages. In most instances, the tumor exceeds 10 cm in diameter by the time it is detected [6,13]. Most feminizing adrenocortical tumor cases described in the literature initially presented with abdominal pain as the primary symptom [14,15].

Gynecomastia in feminizing ACC is primarily related to estrogen excess and subsequent suppression of gonadotropins [6]. In our case, breast enlargement preceded abdominal pain, consistent with most reported cases. Differential diagnosis should nevertheless exclude more common causes such as medication use, chronic liver disease, bronchogenic carcinoma, or testicular disorders [16].

Differential diagnosis should always consider alternative etiologies, including the use of antihypertensive or antidopaminergic medications, digoxin, liver cirrhosis, bronchogenic carcinoma, or other ectopic hormone syndromes, as well as disorders like testicular feminization syndrome [17].

Adrenocortical carcinomas are marked by disordered steroid hormone production, resulting from inconsistent expression patterns of steroidogenic enzymes, which in turn lead to the release of diverse steroid intermediates. Two main pathways have been suggested to account for the elevated estrogen levels seen in feminizing ACCs. The first involves the peripheral conversion of tumor-produced androgens into estrogens by aromatase activity in adipose tissue. The second proposes that this transformation occurs directly within the tumor itself, as supported by the presence of aromatases detected in tumor cells.

The patient and his partner expressed a desire to conceive and reported unprotected sexual intercourse over the past year, without success. Spontaneous conception occurred two months after surgery. It can be hypothesized that the prior infertility was due to suppression of gonadotropin secretion via negative feedback from adrenal origin hyperestrogenism. This hypothesis is supported by the observation that gonadotropin levels remained at the lower limit of the normal range even three months postoperatively.

Within the endocrinology literature, feminizing adrenal tumors in males are typically associated with gynecomastia and hypogonadotropic hypogonadism; however, to date, there are no documented cases of successful conception by the partner following surgical resection.

Regarding the normalization of the hypothalamic–pituitary–gonadal axis, existing medical literature reports a rapid decline in estrogen levels following surgical resection, with normalization of gonadotropins occurring approximately two months postoperatively. As reported by Rich J.M. et al. [5], gonadotropin levels returned to normal approximately two months after surgery—a finding that also observed in our patient. Therefore, it can be concluded that the hypothalamic–pituitary–gonadal axis tends to recover almost completely within approximately three months following curative and recurrence-free surgical excision [5].

Regarding treatment, surgical intervention is recommended for all resectable cases of feminizing adrenocortical carcinoma without evidence of metastasis, as histopathological analysis remains the definitive method for confirming malignancy, and surgery represents the only potential curative approach. A laparoscopic approach is not recommended due to the fragile capsule of FATs and the significant risk of rupture. Open laparotomy is the preferred surgical method [6].

Regarding postoperative adjuvant therapy, a multidisciplinary team was convened to evaluate the available treatment options. In 2017, the research groups led by Massimo Terzolo and Martin Fassnacht published a pivotal study in The New England Journal of Medicine [18], which provided strong evidence supporting the use of mitotane for preventing recurrence in adrenocortical carcinoma. This study established mitotane as the global standard of care for adjuvant therapy, regardless of the presence of risk factors, which were not yet fully defined at that time.

Considering these findings, initiation of mitotane therapy was considered for our patient, but more recent evidence, specifically a clinical trial published in The Lancet Diabetes & Endocrinology in August 2023 by the same group of researchers, challenges the universal need for adjuvant mitotane [19]. Their data suggest that mitotane may be safely omitted in selected low-risk patients, provided three key criteria are met: (1) a complete surgical resection with negative margins (R0), (2) absence of tumor dissemination or advanced staging, and (3) a Ki-67 proliferation index below 10%. In such cases, where the risk of recurrence is minimal, the benefit of adjuvant mitotane is questionable.

Following the establishment of specific criteria to define the low-risk category in adrenocortical carcinoma, the first-ever randomized clinical trial worldwide on adjuvant treatment—ADIUVO—was initiated. This international study enrolled 91 patients across 23 centers in seven countries. Participants with completely resected ACC (R0 resection), stages I–III, and a Ki-67 index of ≤10% were randomized to receive either oral mitotane for two years or active surveillance through regular imaging and laboratory monitoring. The primary endpoint was recurrence-free survival (RFS). At five years, the RFS was 79% in the mitotane group compared to 75% in the surveillance group. Importantly, there was no statistically significant difference in overall survival between the two groups [20].

In our case, these data were presented to the tumor board, and after evaluating the patient’s profile—namely, a completely resected stage T2NxM0 adrenocortical carcinoma with negative margins and a Ki-67 index of 10%—the patient was classified as low risk for recurrence. Consequently, the decision was made to delay the initiation of mitotane therapy in favor of active clinical surveillance. This decision also considered the patient’s preference to postpone adjuvant treatment to attempt conception with his partner.

We present in our article a literature review of all reported cases of estrogen-secreting adrenocortical carcinoma in men between 2015 and 2025, highlighting consistent clinical, biochemical, and histopathologic features (Table 2).

Consistent with our case, gynecomastia was the predominant presenting symptom across all reported cases, often associated with decreased libido, infertility, or testicular atrophy. Preoperative hormonal evaluation consistently demonstrated marked hyperestrogenism, with low or suppressed gonadotropin and testosterone levels—supporting a diagnosis of hypogonadotropic hypogonadism secondary to estrogen excess. Several cases also exhibited co-secretion of adrenal androgens or cortisol, underscoring the functional complexity of these tumors.

Tumor sizes ranged from 4.3 cm to over 21 cm, with a majority located in the right adrenal gland.

Regarding treatment, surgical intervention is recommended for all resectable cases of feminizing adrenocortical carcinoma without evidence of metastasis, as histopathological confirmation of malignancy can only be obtained after resection. A laparoscopic approach is generally not recommended due to the fragile capsule of these tumors and the significant risk of rupture; therefore, open laparotomy is the preferred surgical method. This approach allows for safe manipulation of the tumor and minimizes the risk of peritoneal dissemination.

Following surgery, histopathological and immunohistochemical analyses provide essential information for diagnosis and prognosis. In our case, histopathology confirmed a conventional adrenocortical carcinoma with five positive Weiss criteria, while immunohistochemistry revealed a Ki-67 index of 10% together with positivity for Melan-A, inhibin, calretinin, and focal p53 expression. In the literature, histopathologic analysis of feminizing adrenocortical carcinomas frequently revealed high Weiss scores, and in several instances, Ki-67 indices exceeded 10%, underscoring their malignant potential [21,22,23].

Immunohistochemistry typically demonstrated positivity for aromatase (CYP19A1), inhibin, synaptophysin, and Melan-A. Unlike several reported cases where mitotane was initiated postoperatively, in our patient, the recent ADIUVO trial results supported active surveillance, reflecting a shift in management for low-risk disease.

In line with our findings, postoperative normalization of estradiol and recovery of gonadotropins typically occurred within weeks to months, although persistence of low-normal testosterone, as seen in our patient, has also been described. Our patient’s gynecomastia showed partial regression, a pattern also described in other cases where breast surgery was eventually required to achieve complete resolution. Fertility recovery is rarely documented in the literature, with only isolated cases reporting successful conception. Our case adds to this evidence, as spontaneous conception occurred two months after adrenalectomy.

Despite a generally favorable short-term prognosis following complete tumor resection, long-term outcomes varied. Some patients remained recurrence-free under close surveillance, while others developed local recurrence or distant metastases, necessitating systemic chemotherapy. One case was fatal due to hemorrhagic shock shortly after diagnosis, highlighting the potential for acute complications.

Overall, feminizing ACC remains an exceedingly rare but clinically distinct entity, and its timely recognition is essential to improve oncologic and functional outcomes.

**Table 2:** Summary of all available male cases of feminizing adrenocortical carcinoma with initial presentation as gynecomastia (2015–2025), including one pediatric case.

Author	Year	Age	Clinical Presentation	Tumor Size & Side	Hormonal Profile Preoperative	Treatment	Outcome	Follow-Up
Sykes J et al. [24]	2015	31	Gynecomastia, infertility	9 cm, right adrenal	↑ Estradiol 83 pg/mL; ↑ DHEAS 502 µg/dL; ↓ FSH 0.8 µIU/mL	Open adrenalectomy (Makuuchi incision)	Hormone normalization at 2 weeks; normal gonadotropins at 5 months	Endocrine labs every 6 months, and CT every 6–12 months for at least 5 years
Hatano M. et al. [21]	2016	60	Gynecomastia, right hypochondriac pain, low libido	16 × 11 × 14 cm, right adrenal	↑ Estradiol 284 pg/mL; ↑ DHEAS 560 µg/dL; ↓ FSH 0.2 µIU/mL, ↓ LH 0.1 µIU/mL; ↓ testosterone < 0.6 ng/mL	Open adrenalectomy p0T2N0M0 Weiss 7 Ki-67: 18%	Hormone normalization; later metastases (lymph nodes, peritoneum)	Mitotane was started at 11 months after the discovery of locoregional lymph nodes
Ibrahim F et al. [25]	2018	55	Gynecomastia, testicular hypotrophy, pleuritic left chest pain, hypertension	6.3 cm, right adrenal	↑ Estradiol 134 pg/mL; ↓ Testosterone 109 ng/dL; ↓ FSH/LH	Laparoscopic right adrenalectomy	Not reported	Not reported
Takeuchi et al. [26]	2018	4	Gynecomastia, acute growth spurt	8 cm, right adrenal	↑ Estradiol 28.1 pg/mL; Testosterone 0.82 ng/mL; ↓ LH < 0.1 µIU/mL; ↓ FSH 0.13 µIU/mL; ↑ DHEAS 1950 ng/mL; ↑ Androstenedione 4.6 ng/mL	Chemotherapy (ARAR0332 ^1^ + mitotane) → surgery; Weiss 7; GPOH-MET97 ^2^ postop; hydrocortisone/mineralocorticoid replacement	Gynecomastia resolved; normal growth velocity	No relapse at 2 years
Jeong Y et al. [22]	2019	53	Gynecomastia, abdominal discomfort, right-sided flank pain	21 × 15.3 × 12 cm, right retroperitoneum	↑ Estradiol 820 pg/mL; ↑ DHEAS 578 µg/dL; ↓ FSH 1.07 µIU/mL; ↓ ACTH 4.2 pg/mL; cortisol 16.3 µg/dL; ↑ urinary cortisol 134 µg/24h	Open adrenalectomy + partial hepatectomy pT2N0M0, stage 2, Weiss 6 Ki-67: 20% IHC: inhibin α, MART-1, calretinin	Estradiol ↓ to 70 pg/mL; gynecomastia nearly resolved at 3 months; no recurrence at 21 months	Adjuvant radiotherapy; hydrocortisone replacement
C De Herdt et al. [27]	2019	42	Gynecomastia, jaundice	5.1 cm, right adrenal	↑ Estradiol (44 pg/mL); hypogonadotropic hypogonadism	Open right adrenalectomy pT3L0V0Pn0R0, stage 3 Weiss 4	Estradiol ↓ to 9 pg/mL; gynecomastia resolved; almost complete recovery of the gonadotropic axis after 2 weeks postoperative	Mitotane adjuvant
Gibbons S et al. [23]	2020	52	Gynecomastia, low libido, erectile dysfunction	8 × 8 cm, right adrenal	↑ Estradiol 253.9 pg/mL; ↓ Testosterone 0.7 nmol/L; LH 1.2 IU/L; ↓ FSH 0.1 IU/L	Open right adrenalectomy pT3Nx Ki-67: 60%	Hormone normalization; gynecomastia resolved	Mitotane adjuvant for 2 years, CT at 6 months
Vogt E et al. [28]	2021	58	Gynecomastia, low libido	6.5 cm × 5.2 cm, left adrenal	↑ Estradiol 56.7 pg/mL; ↑ 11-DOC 23.5 nmol/L; ↑ DHEAS 10.6 µmol/L; ↑ Androstenedione 18.1 nmol/L; cortisol excess; low-normal FSH/LH	Laparoscopic adrenalectomy; Weiss 7; Ki-67 5%; IHC: inhibin, synaptophysin, CD31, aromatase (CYP19A1)	Hormone normalization; gynecomastia persisted 1 year after surgery → liposuction planned	Mitotane adjuvant at 8 weeks after resection
Rich J.M. et al. [5]	2023	35	Gynecomastia, low libido, RUQ pain	18 × 8.5 × 14.5 cm, right adrenal	↑ Estradiol 181 pg/mL; ↓ Testosterone 37 ng/dL; ↓ FSH/LH < 0.1 µIU/mL; ↓ ACTH 1 pg/mL; cortisol 8–14.5 µg/dL	Open right adrenalectomy	Hormone normalization after 2 months; Gynecomastia improved and libido recovered	Local recurrence at 6 months-1 cm nodule in the right adrenalectomy area Planned chemotherapy (mitotane or EDP^3^)
Saini J et al. [29]	2023	65	Gynecomastia	4.3 cm, right adrenal, with metastatic lesions after 5 years	↑ Estradiol 72 pg/mL; ↑ Estrone 345 pg/mL; ↑ Progesterone 0.59 ng/mL; ↓ Testosterone 157 ng/dL; ↑ 11-DOC 204 ng/dL; ↑ Renin activity	Initial right laparoscopic adrenalectomy Reintervention-debulking surgery Weiss score 4	Recurrence at 5 years; Reintervention: gynecomastia improved; hormones normalized after 3 months	Mitotane adjuvant + hydrocortisone replacementProgressive disease at 3 months Planned chemotherapy EDP, mitotane was discontinued to reduce the overall toxicity
Abir M et al. [30]	2025	57	Gynecomastia, weight loss, low libido, abdominal pain	Large left adrenal tumor mass	Hyperestrogenism; ↑ 17-OHP, androstenedione, DHEAS	Biopsy externally; IHC: synaptophysin, Melan A	Patient died (hemorrhagic shock)	Not reported

^1^ ARAR0332—Children’s Oncology Group protocol for pediatric adrenocortical tumors; multi-agent chemotherapy including etoposide, doxorubicin, cisplatin, with or without mitotane. ^2^ GPOH-MET97—German Pediatric Oncology Hematology protocol (1997) for adrenocortical tumors in children; mitotane-based chemotherapy regimen. ^3^ EDP—Etoposide, Doxorubicin, Cisplatin regimen.

## 5. Limitations

This case report has several limitations. First, the patient was referred to our endocrinology department after adrenalectomy, which limited the possibility of performing preoperative dynamic endocrine tests such as the dexamethasone suppression test or midnight cortisol evaluation. Second, representative histopathology and immunohistochemistry images were not available for publication, although the diagnosis was rigorously established through detailed histopathological and immunohistochemical examination, confirmed by experienced pathologists.

Finally, as with all rare case reports, the ability to generalize the clinical course and management decisions is limited, underscoring the need for larger datasets and multicenter registries to improve diagnostic and therapeutic strategies for feminizing adrenocortical carcinomas.

## 6. Conclusions

Feminizing adrenocortical carcinoma is a rare entity with high malignant potential and a generally poor prognosis. The present case highlights the importance of a thorough endocrine evaluation in patients presenting with suggestive clinical signs—such as bilateral gynecomastia and infertility—to enable early detection of hormone-secreting adrenal tumors.

The differential diagnosis of gynecomastia in young males should always include rare tumor-related etiologies, especially in the absence of obvious systemic or drug-related causes. The normalization of the hormonal profile and spontaneous conception after surgery suggest that the hypogonadism induced by tumor-related hyperestrogenism may be reversible—an aspect rarely documented in the current medical literature.

Postoperative therapeutic decisions in low-risk cases should be tailored individually, considering emerging evidence supporting active surveillance over universal adjuvant mitotane therapy.

This case contributes to the existing literature by describing a rare presentation of adrenocortical carcinoma associated with gynecomastia and reversible infertility. Given the rarity of such cases, increased awareness and the development of international registries may help refine diagnostic and therapeutic strategies in the future.

## Figures and Tables

**Table 1:** Evolution of hormonal profile.

Laboratory Findings	Preoperative	One Month Postoperative	Three Months Postoperative	Normal Ranges
ACTH	2.6	61.8	114	7.2–63.3 pg/mL
Morning cortisol	12.1	10.1	19.9	4.2–19.6 µg/dL
DHEAS	-	135	128.4	85–690 µIU/mL
Estradiol	90.1	33.7	24.2	29.8–33.1 pg/mL
Testosterone, total	-	2.8	3.1	2.59–8.16 ng/mL
Androstendione	-	168	2.05	0.50–3.50 ng/mL
LH	-	1.2	1.25	1.24–8.62 µIU/mL
FSH	-	2.77	2.72	1.27–19.26 µIU/mL
Plasma Metanephrines	Normal	18.8	25.7	<100 pg/mL
Plasma Normetanephrines	Normal	22.5	70.5	<216 pg/mL
Aldosterone	Normal	5.74	11.3	1.76–23.2 ng/dL
Renin	Normal	13.11	36.19	2.8–39.9 µIU/mL

## Data Availability

All data supporting this case report are contained within the article.

## Abbreviations

The following abbreviations are used in this manuscript

ES-ACCs	Estrogen-secreting adrenocortical carcinoma
17-OHP	17-alpha hydroxyprogesterone
11-DOC	11-deoxycorticosterone
DHEA-S	Dehydroepiandrosterone sulfate
ACTH	Adrenocorticotropic hormone
Ki-67	Proliferation index marker
MEN1	Multiple endocrine neoplasia type 1
FATs	Feminizing adrenocortical tumors
ACCs	Adrenocortical carcinomas
RUQ	Right upper quadrant
IHC	Immunohistochemistry
RFS	Recurrence-free survival
MRI	Magnetic resonance imaging
FSH	Follicle-stimulating hormone
LH	Luteinizing hormone
CT	Computed tomography
US	Ultrasound

## References

[1] Fancellu A., Pinna A., Porcu A. (2014). Feminizing Adrenocortical Carcinoma with Distant Metastases: Can Surgery Be Considered?. Clin. Pract..

[2] Kidd M.T., Karlin N.J., Cook C.B. (2011). Feminizing Adrenal Neoplasms: Case Presentations and Review of the Literature. J. Clin. Oncol..

[3] LaFemina J., Brennan M.F. (2012). Adrenocortical carcinoma: Past, present, and future. J. Surg. Oncol..

[4] Funder J.W., Carey R.M., Mantero F., Murad M.H., Reincke M., Shibata H., Stowasser M., Young W.F. (2016). The Management of Primary Aldosteronism: Case Detection, Diagnosis, and Treatment: An Endocrine Society Clinical Practice Guideline. J. Clin. Endocrinol. Metab..

[5] Rich J.M., Duddalwar V., Cheng P.M., Aron M., Daneshmand S. (2023). Feminizing Adrenocortical Tumor with Multiple Recurrences: A Case Report. Case Rep. Oncol..

[6] Moreno S., Guillermo M., Decoulx M., Dewailly D., Bresson R., Proye C. (2006). Feminizing adreno-cortical carcinomas in male adults. A dire prognosis. Ann. Endocrinol..

[7] Chentli F., Bekkaye I., Azzoug S. (2015). Feminizing adrenocortical tumors: Literature review. Indian. J. Endocrinol. Metab..

[8] Vurallı D., Gönç N., Özön A., Ekinci S., Doğan H.S., Tekgül S., Alikaşifoğlu A. (2022). Feminizing Adrenocortical Tumors as a Rare Etiology of Isosexual/Contrasexual Pseudopuberty. J. Clin. Res. Pediatr. Endocrinol..

[9] Fassnacht M., Assie G., Baudin E., Eisenhofer G., De La Fouchardiere C., Haak H.R., De Krijger R., Porpiglia F., Terzolo M., Berruti A. (2020). Adrenocortical carcinomas and malignant phaeochromocytomas: ESMO–EURACAN Clinical Practice Guidelines for diagnosis, treatment and follow-up. Ann. Oncol..

[10] Allolio B., Fassnacht M. (2006). Adrenocortical Carcinoma: Clinical Update. J. Clin. Endocrinol. Metab..

[11] Lerario A.M., Moraitis A., Hammer G.D. (2014). Genetics and epigenetics of adrenocortical tumors. Mol. Cell Endocrinol..

[12] Phornphutkul C., Okubo T., Wu K., Harel Z., Tracy T.F., Pinar H., Chen S., Gruppuso P.A., Goodwin G. (2001). Aromatase P450 Expression in a Feminizing Adrenal Adenoma Presenting as Isosexual Precocious Puberty. J. Clin. Endocrinol. Metab..

[13] Chentli F., Chabour F., Bouchibane D., Nouar N. (2017). Feminizing Adrenocortical Carcinoma Without Gynecomastia. Oman Med. J..

[14] Fukai N., Hirono Y., Yoshimoto T., Doi M., Ohtsuka Y., Homma K., Shibata H., Sasano H., Hirata Y. (2006). A Case of Estrogen-secreting Adrenocortical Carcinoma with Subclinical Cushing’s Syndrome. Endocr. J..

[15] Visconti E.B., Peters R.W., Cangir A., Zorn G.L., Fisher S. (1978). Unusual case of adrenal cortical carcinoma in a female infant. Arch. Dis. Child..

[16] Narula H.S., Carlson H.E. (2014). Gynaecomastia—Pathophysiology, diagnosis and treatment. Nat. Rev. Endocrinol..

[17] Lanigan D., Choa R.G., Evans J. (1993). A feminizing adrenocortical carcinoma presenting with gynaecomastia. Postgrad. Med. J..

[18] Terzolo M., Angeli A., Fassnacht M., Daffara F., Tauchmanova L., Conton P.A., Rossetto R., Buci L., Sperone P., Grossrubatscher E. (2007). Adjuvant Mitotane Treatment for Adrenocortical Carcinoma. N. Engl. J. Med..

[19] Terzolo M., Fassnacht M., Perotti P., Libé R., Kastelan D., Lacroix A., Arlt W., Haak H.R., Loli P., Decoudier B. (2023). Adjuvant mitotane versus surveillance in low-grade, localised adrenocortical carcinoma (ADIUVO): An international, multicentre, open-label, randomised, phase 3 trial and observational study. Lancet Diabetes Endocrinol..

[20] News Medical (2023). Study Shows Not All Adrenal Carcinoma Patients Need Mitotane After Surgery. https://www.news-medical.net/news/20230825/Study-shows-not-all-adrenal-carcinoma-patients-need-mitotane-after-surgery.aspx.

[21] Hatano M., Takenaka Y., Inoue I., Homma K., Hasegawa T., Sasano H., Awata T., Katayama S. (2016). Feminizing Adrenocortical Carcinoma with Distinct Histopathological Findings. Intern. Med..

[22] Jeong Y., Cho S.C., Cho H.J., Song J.S., Kong J.S., Park J.W., Ku Y.H. (2019). Estrogen-secreting adrenocortical carcinoma. Yeungnam Univ. J. Med..

[23] Gibbons S.M., Jassam N., Abbas A., Stuart K., Fairhurst A., Barth J.H. (2020). Gynaecomastia caused by a feminizing adrenal tumour. Ann. Clin. Biochem. Int. J. Lab. Med..

[24] Sykes J., Ellis J.L., Bukavina L., Koch C.A., Wei S., Kutikov A. (2022). Estradiol-secreting adrenal oncocytoma in a 31-year old male. Urol. Case Rep..

[25] Ibrahim F. (2018). Male gynecomastia: A rare case of feminizing adrenal cortical carcinoma. J. Hosp. Med..

[26] Takeuchi T., Yoto Y., Ishii A., Tsugawa T., Yamamoto M., Hori T., Kamasaki H., Nogami K., Oda T., Nui A. (2018). Adrenocortical carcinoma characterized by gynecomastia: A case report. Clin. Pediatr. Endocrinol..

[27] De H.C., Philipse E., De B.C. (2019). An adrenal tumor with gynecomastia. Endocr. Abstr..

[28] Vogt E.C., Hammerling K., Sorbye H., Heie A., Sulen A., Ueland G., Husebye E., Methlie P. (2021). Feminizing adrenal tumor identified by plasma steroid profiling. Endocrinol. Diabetes Metab. Case Rep..

[29] Saini J., Navin P., Rivera M., Bancos I. (2023). Gynecomastia in a Man with Adrenal Mass. JCEM Case Rep..

[30] Abir M. (2025). Bilateral gynecomastia revealing adrenocorticaloma. Endocr. Abstr..

